# Glycemic penalty index for adequately assessing and comparing different blood glucose control algorithms

**DOI:** 10.1186/cc6800

**Published:** 2008-02-26

**Authors:** Tom Van Herpe, Jos De Brabanter, Martine Beullens, Bart De Moor, Greet Van den Berghe

**Affiliations:** 1 Katholieke Universiteit Leuven, Department of Electrical Engineering (ESAT), Research Division SCD, Kasteelpark Arenberg 10, B-3001 Leuven (Heverlee), Belgium; 2 Hogeschool KaHo Sint-Lieven (Associatie K.U. Leuven), Dept. Industrieel Ingenieur, Gebroeders Desmetstraat 1, B-9000 Gent, Belgium; 3 Leuvens Universitair Dienstencentrum voor Informatica en Telematica (LUDIT), W. de Croylaan 52a, B-3001 Leuven (Heverlee), Belgium; 4 Katholieke Universiteit Leuven, Department of Intensive Care Medicine, University Hospital Gasthuisberg, Herestraat 49, B-3000 Leuven, Belgium

## Abstract

**Introduction:**

Blood glucose (BG) control performed by intensive care unit (ICU) nurses is becoming standard practice for critically ill patients. New (semi-automated) 'BG control' algorithms (or 'insulin titration' algorithms) are under development, but these require stringent validation before they can replace the currently used algorithms. Existing methods for objectively comparing different insulin titration algorithms show weaknesses. In the current study, a new approach for appropriately assessing the adequacy of different algorithms is proposed.

**Methods:**

Two ICU patient populations (with different baseline characteristics) were studied, both treated with a similar 'nurse-driven' insulin titration algorithm targeting BG levels of 80 to 110 mg/dl. A new method for objectively evaluating BG deviations from normoglycemia was founded on a smooth penalty function. Next, the performance of this new evaluation tool was compared with the current standard assessment methods, on an individual as well as a population basis. Finally, the impact of four selected parameters (the average BG sampling frequency, the duration of algorithm application, the severity of disease, and the type of illness) on the performance of an insulin titration algorithm was determined by multiple regression analysis.

**Results:**

The glycemic penalty index (GPI) was proposed as a tool for assessing the overall glycemic control behavior in ICU patients. The GPI of a patient is the average of all penalties that are individually assigned to each measured BG value based on the optimized smooth penalty function. The computation of this index returns a number between 0 (no penalty) and 100 (the highest penalty). For some patients, the assessment of the BG control behavior using the traditional standard evaluation methods was different from the evaluation with GPI. Two parameters were found to have a significant impact on GPI: the BG sampling frequency and the duration of algorithm application. A higher BG sampling frequency and a longer algorithm application duration resulted in an apparently better performance, as indicated by a lower GPI.

**Conclusion:**

The GPI is an alternative method for evaluating the performance of BG control algorithms. The blood glucose sampling frequency and the duration of algorithm application should be similar when comparing algorithms.

## Introduction

Hyperglycemia and insulin resistance are common in critically ill patients (even those without diabetes mellitus [1-3]) and are associated with adverse outcome in a variety of clinical settings [4-7]. In two randomized controlled studies of mechanically ventilated patients admitted to a surgical and a medical intensive care unit (ICU), normalization of blood glucose (BG) (between 80 and 110 mg/dl) with insulin significantly reduced morbidity and mortality rates [8,9]. BG control, aiming at normoglycemia, is now attempted in ICUs worldwide. This is usually performed by nurses or physicians who are instructed by 'manual' guidelines or algorithms [10-14]. These algorithms are developed with the purpose of determining the insulin dose that is required to obtain normoglycemia based on intermittent BG readings. Computer-based protocols (as presented in [15-27]) have the potential to facilitate and improve glycemic control and to reduce the workload for medical staff. However, these 'new' protocols require stringent validation before they can replace the currently existing 'nurse-driven' insulin protocols.

Three types of methods exist for evaluating the adequacy of insulin titration algorithms. All of them, however, show weaknesses that may lead to erroneous conclusions. The first method simply computes the average of all BG readings. In spite of its popularity, it must be stressed that normoglycemia can be falsely assumed even in the presence of severely abnormal BG values. Indeed, hypoglycemic and hyperglycemic events can artificially lower or raise, respectively, the calculated average and can even balance each other, leading to an apparently 'normal' average BG.

A second method comprises single measurements; for example taking BG readings at a fixed time of day, the minimum/maximum BG values, and the time needed to reach the target BG. Alternative single measurements count the number of hypoglycemic or hyperglycemic events. Although such measurements are useful, they do not capture the BG dynamics.

Recently, the hyperglycemic index (HGI) was presented as a third, more advanced, tool for assessing glucose control (in the ICU) with respect to hyperglycemic events [28]. The HGI is defined as the area under the glucose curve above 6.0 mmol/l (108 mg/dl) divided by the length of ICU stay. Two conditions to be satisfied before applying the HGI were proposed [29]. First, the number of BG measurements should be sufficiently high; ideally a near-continuous glucose read-out. Second, the considered sampling frequency should be comparable in both patient groups when comparing the adequacy of two insulin titration algorithms. It is important to note that area-under-the-curve methods (such as the HGI) currently rely on the assumed (linear) relationship between intermittent BG readings, since no reliable and accurate near-continuous glucose sensor is presently available [15,19,30].

Another critical point of this technique is that outliers can potentially warp the obtained results due to the possible presence of extreme (hyperglycemic) observations that may have an impact on the computed area-under-the-curve. This is an important feature when realizing that sensor accuracy (and reliability) typically decreases as the BG level increases [31-33]. It is clear that the presence of outliers also affect the computed average BG values (see Materials and methods). Finally, HGI only transforms the hyperglycemic, and not the hypoglycemic, glucose dynamics into a number. Of course, we acknowledge that the design of an alternative hypoglycemic index (as already suggested in [34]) would overcome this last aspect.

The aim of the current study was to design a tool for adequate comparison of BG control algorithms. In the first part of the study, we developed a grading system that scores normal, hypoglycemic, and hyperglycemic BG readings; the glycemic penalty index (GPI). In the second part of the study, the performance of the GPI was compared on an individual as well as a population basis with the current standard evaluation methods (average morning BG, mean of all BG readings and HGI), using data from a selected set of patients. In the third part of the study, we investigated the importance or the weight of four clinically selected parameters (BG sampling frequency, duration of algorithm application, severity of disease, and type of illness) on GPI.

## Materials and methods

### Mathematical computation of GPI

**Figure 1:**
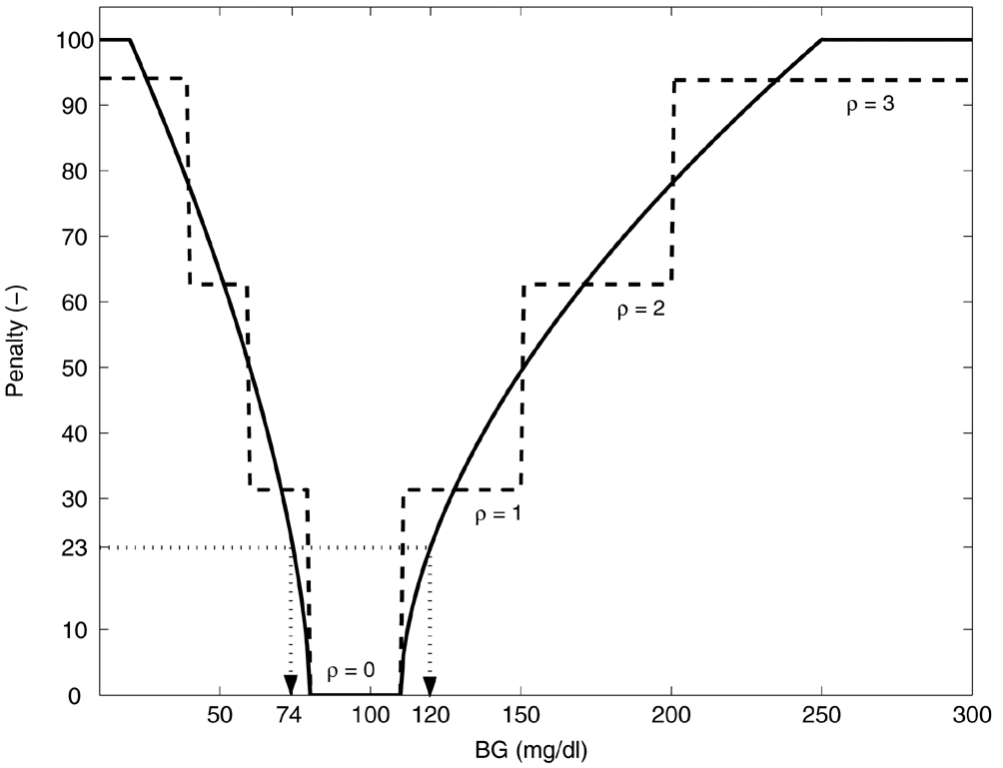
Penalty index as a function of blood glucose (BG). Each BG observation corresponds to a penalty. The dashed line represents the staircase penalty index function (here, the penalty is denoted as ρ). For reasons explained in the text, this staircase function was transformed into a more smoothed penalty index function, which is illustrated by the solid line. The penalties are symbolized by β, γ, and δ for the low, normal, and high BG measurements, respectively. The 'clinically acceptable' cut-off glycemic penalty index (GPI) equals 23 and corresponds to a 'clinically acceptable' BG range of 74–120 mg/dl. The target normoglycemic range, however, remains 80 to 110 mg/dl with a corresponding penalty value equal to 0.

We defined the GPI as a tool that scores BG readings in order to evaluate the overall BG dynamics obtained in the considered patient by applying a specific ICU insulin titration algorithm. The computation of the GPI used a penalty strategy that was based on clinical 'expert' knowledge. The glycemic target range in the ICU was defined as 80 to 110 mg/dl [8,9], with a penalty value for all BG values lying in this range, therefore set at 0. Hyperglycemic and hypoglycemic events were amplified (in terms of the assigned penalties) in relation to the magnitude of their deviation from the target range. Table 1 gives an overview of the glycemic threshold values that are generally accepted for use in the ICU. Each glycemic range was associated with a penalty ρ leading to a staircase 'expert' penalty function when considering the full glycemic range (see Figure 1, dashed line).

**Table 1:** Penalty evaluation strategy showing threshold values and penalty values for the evaluation of blood glucose (BG) control in the intensive care unit (ICU)

**Range no.**	**Glycemic range (mg/dl)**	**Clinical description**	**Penalty (ρ)**	**Reference**
1	*BG *< 40	Hypoglycemic alarm	3	[35]
2	40 ≤ *BG *< 60	Hypoglycemia	2	[35]
3	60 ≤ *BG *< 80	Slight hypoglycemia	1	[35]
4	80 ≤ *BG *≤ 110	Normoglycemia	0	[8, 9]
5	110 <*BG *≤ 150	Slight hyperglycemia	1	[30]
6	150 <*BG *≤ 200	Hyperglycemia	2	[30]
7	200 <*BG*	Hyperglycemic alarm	3	[8]

We then smoothed this staircase function in order to avoid abrupt changes in the penalty function. However, the clinically accepted normoglycemic target range, the hypoglycemic alarm level (i.e., BG values below 40 mg/dl [35]), and the hyperglycemic alarm level (i.e., BG values above 200 mg/dl [8]) were respected in the design of the more smoothed function. This gave the advantage that penalties were gradually increasing as a function of the increasing deviation from the target range. Accordingly, BG measurement errors caused by sensor inaccuracies and methodology inaccuracies due to sampling handling only had a limited impact on the overall assessment of a BG algorithm.

The smooth penalty function was subsequently optimized by designing a polynomial function in the BG ranges 20 to 79 mg/dl and 111 to 250 mg/dl. The squared differences between the staircase and the more smoothed function were minimized by applying ordinary least squares [36]. The penalty index corresponding to the normoglycemic range (80 to 110 mg/dl) was set at 0. BG values lower than 20 mg/dl and higher than 250 mg/dl were assigned a maximum value to avoid that outliers would distort the obtained GPI (as can be the case with currently used evaluation methods; see above).

### Comparison of GPI with currently used evaluation methods

The average morning BG, the average BG (i.e., the mean of all BG readings), the HGI, and the GPI were computed for each patient in the study set. Though the glycemic target range was 80 to 110 mg/dl, we defined 120 mg/dl as a 'clinically acceptable' upper limit taking into account possible sensor inaccuracies and methodology inaccuracies due to sampling handling [37-41]. Therefore, the cut-off values for evaluating the performance of the BG algorithm were arbitrarily set as follows. Average morning BG readings below 120 mg/dl, average BG values below 120 mg/dl, and HGIs below 12 mg/dl (i.e., an average hyperglycemic value below 108+12 = 120 mg/dl) were labeled 'clinically acceptable'. The cut-off GPI that explained whether the insulin titration algorithm was acceptable or not was determined by entering 120 mg/dl, as cut-off BG, to the developed smooth penalty function. Next, the performance of the existing standard evaluation methods and the GPI were compared both on individual and population basis.

### Study procedure and patient population

We assembled two different data sets obtained from patients who had been admitted to the surgical ICU division of the University Hospital KU Leuven (Belgium) and who had been treated by the same nursing team but for whom a different BG sampling frequency was used. Whole BG in undiluted arterial blood was measured by means of the same glucose analyzer (ABL700 Radiometer Medical, Copenhagen) in both patient groups. The first patient group comprised 41 subjects (patient group 1) who were retrospectively selected from the data originally described in [8]. They were chosen to cover variable demographics (Table 2) and durations of stay in the ICU. The goal was to retrieve a representative sample for the larger patient group of [8] in terms of duration of intensive care and proportion of diagnostic subgroups. The BG sampling frequency and insulin titration guidelines (as described in [11]) were identical for all patients. The second patient group comprised 52 subjects (patient group 2) with variable demographics and duration of stay in the ICU, of whom only the first 2 days were considered, during which the sampling frequency was set at once every hour. Only those patients who were expected to have a duration of stay of more than 2 days were selected for this study. The titration was performed by the same nursing staff, who followed the same guidelines, as used for patient group 1.

**Table 2:** Patient populations 1 and 2 (both coming from a surgical intensive care unit (ICU)) showing characteristics of both surgical ICU patient groups

**Variable**	**Patient group 1**	**Patient group 2**
No. of patients	41	52
Male sex, no. (%)	27 (65.8)	29 (55.8)
Age, years (SD)	59.8 (17.6)	65.2 (16.1)
Body mass index, kg/m^2 ^(SD)	27.0 (5.2)	25.1 (4.7)
Reason for intensive care, no. (%):		
Cardiac surgery, type 1	11 (26.8)	32 (61.5)
Non-cardiac indication:	30 (73.2)	20 (38.5)
Multiple trauma or severe burns, type 2	7 (17.1)	0 (0)
Neurologic disease, cerebral trauma, or complicated brain surgery, type 3	4 (9.8)	2 (3.9)
Complicated lung or esophageal thoracic surgery, respiratory insufficiency, or both, type 4	7 (17.1)	5 (9.6)
Complicated abdominal surgery or peritonitis, type 5	5 (12.2)	10 (19.2)
Transplantation, type 6	3 (7.3)	2 (3.9)
Complicated vascular surgery, type 7	2 (4.9)	0 (0)
Other, type 8	2 (4.9)	1 (1.9)
APACHE II score (first 24 h) (SD)	11 (6)	16 (4)
Mean BG – mg/dl (SD):	108 (37)	104 (29)
Minimal BG, mg/dl	37	37
Maximal BG, mg/dl	379	307

SD, standard deviation.

Except for the different BG sampling frequency and the duration of algorithm application, both patient groups varied for type of illness and the APACHE II (Acute Physiology and Chronic Health Evaluation; [42]) score. The average APACHE II score was higher in group 2. The differences between the patient groups allowed us to analyze the influence (weight) of the four selected clinically relevant parameters (see below) on GPI in an appropriate way. Informed consent was obtained from the closest family member at ICU admission. The study protocol was approved by the Institutional Ethical Review Board.

### Definition of parameters

Four different parameters were selected based on their clinically expected influence on GPI. The first parameter was the average BG sampling frequency (*f*), which was the average number of BG readings (per time unit) that were available and used by the insulin titration algorithm. The conversion to time dimension was realized by taking the inverse of the frequency (e.g., *f *= 0.5 h^-1 ^corresponds to a time interval of 2 h). The second parameter was the duration of algorithm application (*D*), which was the time period that the control algorithm was effectively used for a given patient. The next parameter was the severity of disease (*A*), scored by the APACHE II score, still the most reported and used system in ICU and, therefore, the one selected for this study (although more recent scoring systems may perform better at grading severity of illness). The APACHE II score of the first 24 h after admission to the ICU was calculated for each patient using parameters of acute physiology and chronic healthcare. The final parameter under study was the type of illness. As an example, eight reasons for admission to the ICU were considered in this analysis: cardiac surgery (type 1), multiple trauma or severe burns (type 2), neurologic disease, cerebral trauma or complicated brain surgery (type 3), complicated lung or esophageal thoracic surgery, respiratory insufficiency, or both (type 4), complicated abdominal surgery or peritonitis (type 5), transplantation (type 6), complicated vascular surgery (type 7), and others (type 8).

### Statistics

The Kruskal-Wallis test was used for comparing the medians of two or more groups of data. Depending on the distribution of the residuals, general and generalized linear models were built. In the general linear model, the Shapiro-Wilk test was applied for testing the normality of the residuals. The determination of the significance (weight) of the specific parameter on GPI (i.e., 'input selection' for the model) was based on *F*-tests for the general linear model and the likelihood ratio Chi-square statistics for the generalized linear model. For the last type of model, Wald statistics were used. Pearson's correlation coefficients (*R*) were calculated for quantifying the relation between continuous variables. In all applied tests p values < 0.05 were considered to be significant.

## Results

### Mathematical computation of GPI

The clinically defined staircase penalty function was transformed to a more smoothed penalty function. The obtained function was mathematically formulated as follows:

For time step *t *= 1 to *N*_*total*_:

*BG*_*t *_< 20 mg/dl: β_*i *_= 100,

20 mg/dl ≤ *BG*_*t *_< 80 mg/dl: β_*i *_= 7.4680 (80 – *BG*_*t*_)^0.6337^,

80 mg/dl ≤ *BG*_*t *_≤ 110 mg/dl: γ_*j *_= 0,

110 mg/dl <*BG*_*t *_≤ 250 mg/dl: δ_*k *_= 6.1767 (*BG*_*t *_– 110)^0.5635^,

250 mg/dl <*BG*_*t*_: δ_*k *_= 100

where β_*i *_was the penalty index for a glucose reading *BG*_*t *_of the hypoglycemic range (i.e., *BG*_*t *_< 80 mg/dl), γ_*j *_for the normoglycemic range (i.e., 80 mg/dl ≤ *BG*_*t *_≤ 110 mg/dl), and δ_*k *_for the hyperglycemic range (i.e., *BG*_*t *_> 110 mg/dl). The indices *i*, *j*, and *k *were used to count the number of hypoglycemic, normoglycemic, and hyperglycemic events, respectively. The symbol that represents the number of BG measurements in the full glycemic range (that were available for the considered patient) was *N*_*total*_. This more smoothed function is illustrated in Figure 1 (solid line).

All BG values from a patient corresponded to specific penalty values as directly followed from the smoothed function. Next, the GPI was calculated for each patient:

$$\text{GPI}=\frac{\sum_{i=1}^{N_{Hypo}}\beta_{i}+\sum_{k=1}^{N_{Hyper}}\delta_{k}}{N_{Total}}$$

where *N*_*Hypo *_is the symbol for the number of BG measurements in the hypoglycemic range, and *N*_*Hyper *_the symbol for the hyperglycemic range. The relative contribution of the hypoglycemic values to GPI (denoted as *C*_*Hypo*_) was determined as follows:

$$C_{Hypo}=\frac{\sum_{i=1}^{N_{Hypo}}\beta_{i}}{\sum_{i=1}^{N_{Hypo}}\beta_{i}+\sum_{k=1}^{N_{Hyper}}\delta_{k}}100\%$$

Analogously, the relative contribution of the hyperglycemic values to GPI (denoted as *C*_*Hyper*_) was computed as follows:

$$C_{Hyper}=\frac{\sum_{k=1}^{N_{Hyper}}\delta_{k}}{\sum_{i=1}^{N_{Hypo}}\beta_{i}+\sum_{k=1}^{N_{Hyper}}\delta_{k}}100\%$$

### Comparison of GPI with currently used methods

The 'clinically acceptable' upper limit BG (120 mg/dl) was entered into the above developed smoothed penalty function, giving 23 as 'clinically acceptable' cut-off GPI. Next, the inverse smoothed penalty function was used to compute the lower limit BG that corresponded to GPI = 23 (Figure 1). The 'clinically acceptable' BG range was found to be 74 to 120 mg/dl but the glycemic target range remained at 80 to 110 mg/dl. In other words, a computed GPI below 23 allowed us to conclude that the insulin titration algorithm was able to control BG according to the clinical requirements. Ideally, however, all BG readings should fall within the 80 to 110 mg/dl zone leading to a GPI equal to 0.

**Figure 2:**
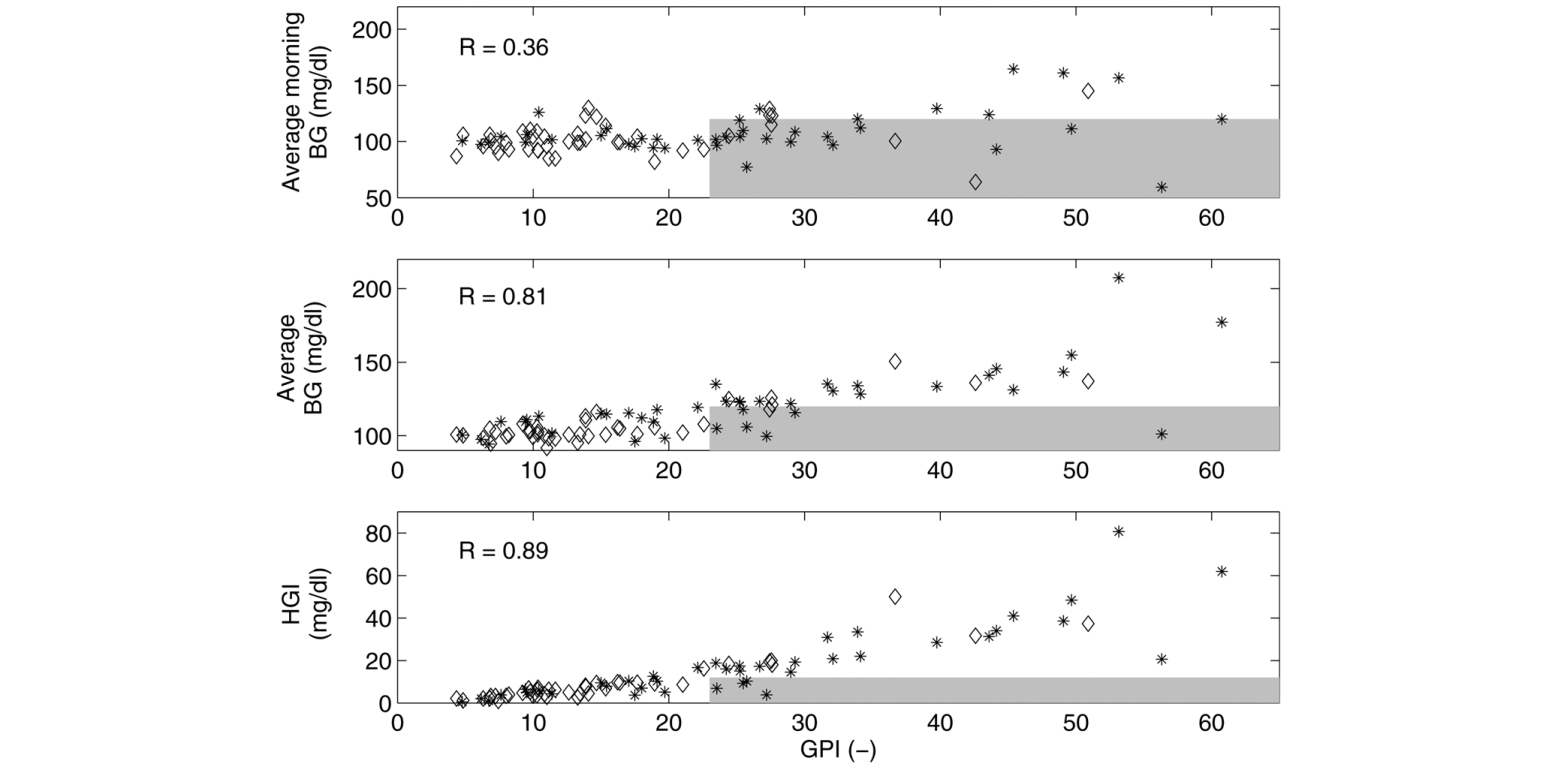
Standard evaluation techniques versus glycemic penalty index (GPI) for patient group 2 and 1 (but considering only the data of the first 48 h in the latter). The results of the standard evaluation methods are plotted against the results of the new proposed assessment tool (GPI). The top panel shows the average morning blood glucose (BG) readings as a function of GPI. The middle panel represents the average BG values versus the GPI values. Finally, the bottom panel illustrates the computed HGI values as a function of GPI. The shaded area contains those patients whose BG profile was evaluated differently: 'clinically acceptable' for the standard measures, 'clinically unacceptable' for GPI. The stars denote the patients from group 1 whereas the diamonds represent the patients from group 2. The correlation coefficients (*R*) for the existing measures with respect to GPI are depicted in the respective panels.

**Figure 3:**
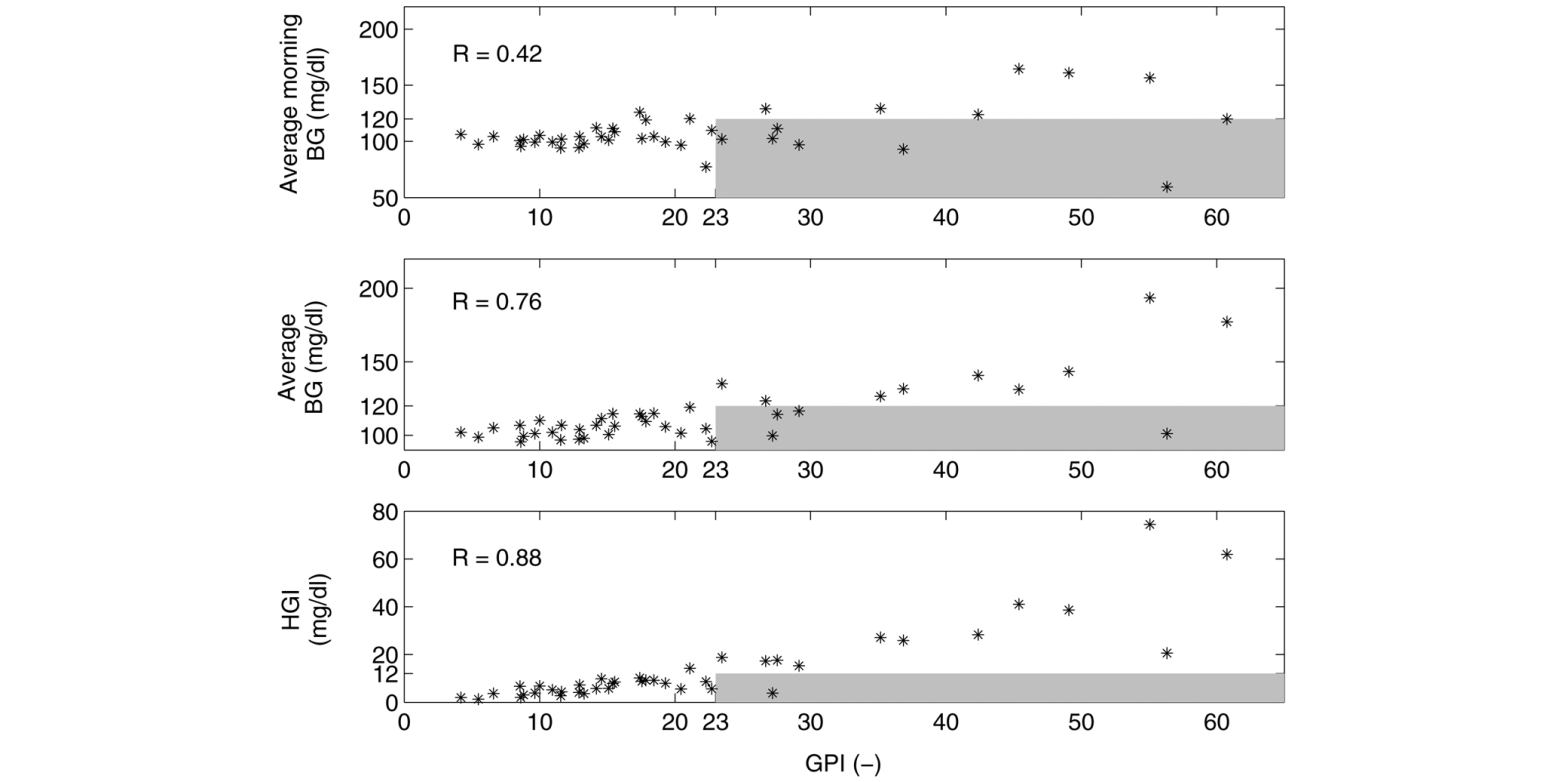
Standard evaluation techniques versus glycemic penalty index (GPI) for patient group 1 (considering the full dataset). The blood glucose (BG) profiles of the patients of group 1 are evaluated by applying the same standard techniques as mentioned in Figure 2 and are again presented as a function of GPI. The patients that belong to the shaded area got a different algorithm evaluation dependent on the method that was used (respective 'standard' method versus GPI). The shaded area contains those patients whose BG profile was evaluated differently: 'clinically acceptable' for the standard measures, 'clinically unacceptable' for GPI.

**Figure 4:**
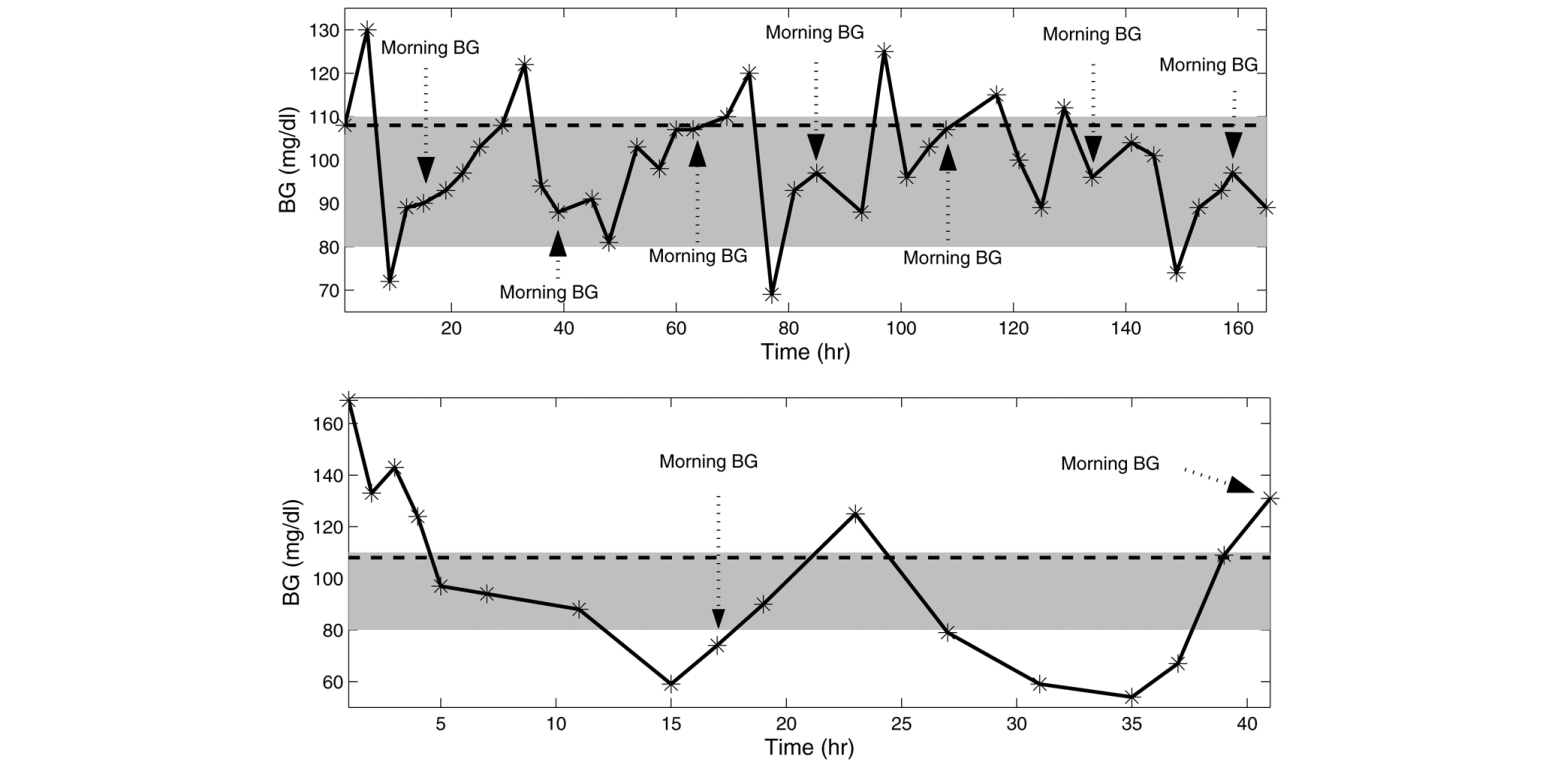
Measured blood glucose (BG) signals of patient no. 19 and 11. The measured BG readings (ABL700 Radiometer Medical) of patient no. 19 (top panel) and 11 (bottom panel) from group 1 are represented by stars. These BG profile examples denote tight glycemic control for patient no. 19 but rather poor glycemic control for patient no. 11. It is important to note that different time scales were used as patient no. 11 stayed in the intensive care unit (ICU) for only a short time period. Further, the obtained BG measurements were linearly interpolated. The normal BG range (target range) is indicated by the shaded area (80–110 mg/dl). The hyperglycemic index (HGI) is the area under the glucose curve above 6 mmol/l (108 mg/dl, as illustrated by the dashed line [28]). The morning BG values are indicated by the dotted arrows. Table 4 shows all computed measures in detail. Based on clinical expert knowledge, it can be observed that the assessment of the BG profile of patient no. 11 using the glycemic penalty index (GPI) is different from the evaluation with average (morning) BG and HGI.

Table 3 gives a detailed overview of the results of the evaluation methods (average morning BG, average BG, HGI, GPI, and the relative contribution of the low (*C*_*hypo*_) and high (*C*_*hyper*_) BG observations to the computed GPI) that are applied to patient group 1. A summary of this is presented in Table 4. Figures 2 and 3 further summarize the performance differences between the evaluation methods applied to the individual patients. The correlation coefficients for the already existing measures with respect to GPI are depicted in each respective panel. Finally, Figure 4 illustrates the BG profile of patient no. 19 (top panel, no misleading effect of standard assessment methods), whose BG was tightly controlled, and patient no. 11 (bottom panel, assessment misled by average (morning) BG and HGI) with poor BG control (see also Table 4 for exactly computed measures).

**Table 3:** Blood glucose (BG) control assesment; evaluation of BG control by computing the average morning BG, the average BG, the hyperglycemic index (HGI), and the glycemic penalty index (GPI) for patient group 1

**Patient no.**	**Average morning BG (mg/dl)**	**Average BG (mg/dl)**	**HGI (mg/dl)**	**GPI (*C*_*Hypo*_(%) – *C*_*Hyper*_(%))**
1	161	143	39	49 (16.2 – 83.8)
2	129	123	17	27 (4.0 – 96.0)
3	124	141	28	42 (9.6 – 90.4)
4	165	131	41	45 (23.0 – 77.0)
5	97	101	6	20 (34.7 – 65.3)
6	77	105	9	22 (36.6 – 63.4)
7	104	115	9	18 (4.2 – 95.8)
8	129	127	27	35 (18.4 – 81.6)
9	93	132	26	37 (29.5 – 70.5)
10	109	106	8	16 (23.5 – 76.5)
11	103	100	4	27 (49.8 – 50.2)
12	97	117	15	29 (21.4 – 78.6)
13	100	101	4	10 (31.2 – 68.8)
14	103	113	9	18 (11.5 – 88.5)
15	98	98	4	13 (47.5 – 52.5)
16	111	114	18	28 (27.5 – 72.5)
17	101	101	6	15 (40.0 – 60.0)
18	104	105	4	7 (1.7 – 98.3)
19	97	99	1	5 (39.0 – 61.0)
20	102	99	3	9 (25.7 – 74.3)
21	102	107	4	12 (16.2 – 83.8)
22	126	115	10	17 (11.9 – 88.1)
23	60	101	21	56 (59.7 – 40.3)
24	102	135	19	23 (0 – 100)
25	101	107	7	9 (10.3 – 89.7)
26	100	106	8	19 (29.7 – 70.3)
27	112	107	6	14 (27.8 – 72.2)
28	104	111	10	15 (1.3 – 98.7)
29	105	110	7	10 (0 – 100)
30	120	177	62	61 (5.2 – 94.8)
31	110	96	6	23 (57.1 – 42.9)
32	99	102	5	11 (18.3 – 81.7)
33	120	119	14	21 (12.1 – 87.9)
34	96	96	2	9 (51.2 – 48.8)
35	94	97	3	12 (48.0 – 52.0)
36	157	194	74	55 (19.5 – 80.5)
37	94	97	4	13 (49.1 – 50.9)
38	106	102	2	4 (10.6 – 89.4)
39	104	104	7	13 (24.2 – 75.8)
40	112	115	8	15 (6.9 – 93.1)
41	119	109	9	18 (18.0 – 82.0)
				
Mean (SD)	108 (20)	114 (21)	14 (16)	22 (14)
Median (25% to 75% IQR)	104 (98 to 112)	107 (101 to 116)	8 (4 to 17])	18 (12 to 27)
				*C*_*Hypo*_: 21.4 (10.3 to 34.7)
				*C*_*Hyper*_: 78.6 (63.4 to 89.4)

IQR, interquartile range; SD, standard deviation.

**Table 4:** Blood glucose (BG) control assessment for a subset of patient group 1 (summary of Table 3); evaluation of BG control by computing the average morning BG, the average BG, the hyperglycemic index (HGI), and the glycemic penalty index (GPI) for a subset of patient group 1

**Patient no.**	**Average morning BG (mg/dl)**	**Average BG (mg/dl)**	**HGI (mg/dl)**	**GPI (*C*_*Hypo *_(%) – *C*_*Hyper *_(%))**
1	161	143	39	49 (16.2 – 83.8)
11	103	100	4	27 (49.8 – 50.2)
19	97	99	1	5 (39.0 – 61.0)
				
Mean (SD)	108 (20)	114 (21)	14 (16)	22 (14)
Median (25% to 75% IQR)	104 (98 to 112)	107 (101 to 116)	8 (4 to 17)	18 (12 to 27)
				*C*_*Hypo*_: 21.4 (10.3 to 34.7)
				*C*_*Hyper*_: 78.6 (63.4 to 89.4)

In most studies, however, the BG control algorithm is evaluated using the patient population rather than on individual patients [8,9,20,21,27]. The population results for patient group 1 are mentioned in Table 4. The most appropriate way to present the population HGI and GPI values is by calculating the median and 25% to 75% interquartile (IQ) range as these data were not normally distributed.

### Weight determination for the selected parameters

**Figure 5:**
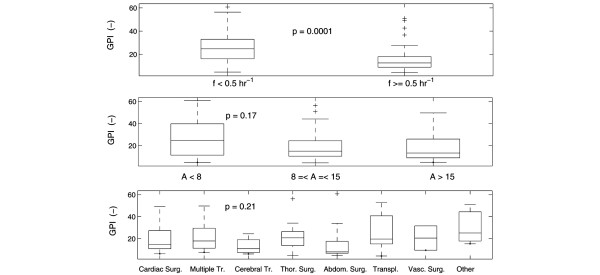
Univariate relationship between patient groups 2 and 1 (but considering only the data of the first 48 h in the latter). Univariate relationship (expressed in box plots) between average BG sampling frequency (*f*) and GPI (top panel), APACHE II score (*A*) and glycemic penalty index (GPI) (middle panel), and type of illness and GPI (bottom panel) for patient group 2 and 1 (but considering only the data of the first 48 h in the latter). The p values of the null hypothesis that the GPI medians per group are equal are mentioned in each panel. A significant difference was found for the average blood glucose (BG) sampling frequency: the BG profiles with *f *≥ 0.5 h^-1 ^(i.e., time intervals less than 2 h) are related to stricter glycemic control (lower GPI).

The impact of the variables under study on GPI for the patients belonging to patient group 2 and 1 (but considering only the data of the first 48 h in the latter) is illustrated in Figure 5. As the duration of algorithm application was set at 48 h, this variable was not included in this analysis. The p values of the null hypothesis that the GPI medians per group are equal are noted in each respective panel (only significant inequality for the average BG sampling frequency). Multiple regression analysis on these data revealed that the average BG sampling frequency was the only parameter that significantly (p = 0.0051) impacted the assessment of insulin titration algorithms: an inversely proportional effect was observed.

**Figure 6:**
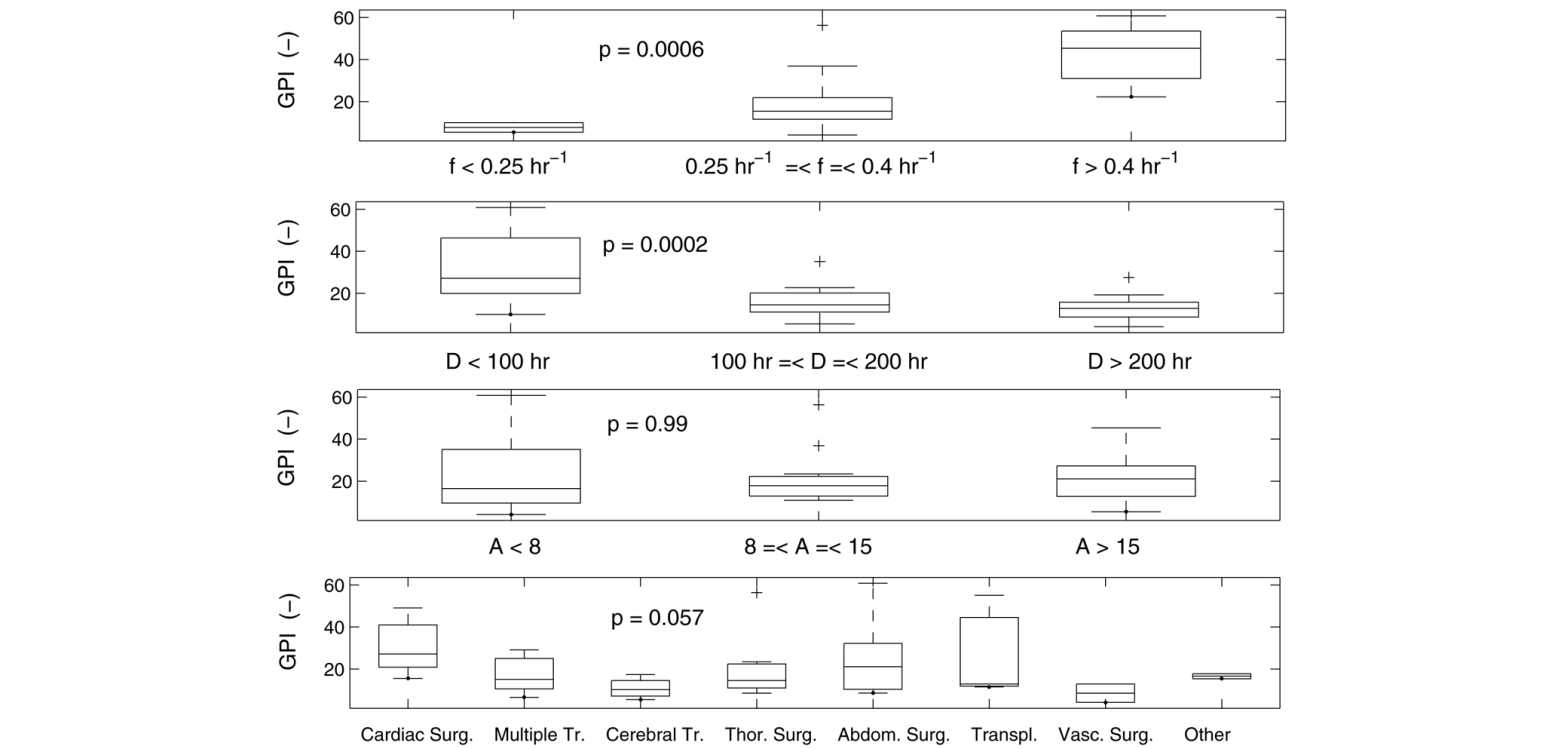
Univariate relationships in patient group 1. Univariate relationship (expressed in box plots) between average blood glucose (BG) sampling frequency (*f*) and glycemic penalty index (GPI) (top panel), duration of algorithm application (*D*) and GPI (second panel), APACHE II score (*A*) and GPI (third panel), and type of illness and GPI (bottom panel) based on patient group 1. The p values of the null hypothesis that the GPI medians per group are equal are mentioned in each panel. A significant difference was found for the average BG sampling frequency and the duration of algorithm application. The longer the algorithm is applied to the patient and the longer the time intervals between successive measurements, the tighter the glycemic control (lower GPI). The apparently contradictory impact of the BG sampling frequency on GPI can be explained by the negative correlation between the variables duration of algorithm application and average BG sampling frequency (see text).

Figure 6 illustrates the independent impact of all four variables under study on GPI for patient group 1 (considering all the available data of this group). The obtained p values of the null hypothesis that the GPI medians per group are equal are again noted in the respective panels (significant inequality for the average BG sampling frequency and duration of algorithm application). Multiple regression analysis returned that both duration of algorithm application (p = 0.032) and the product of duration of algorithm application and average BG sampling frequency (p = 0.025) significantly influenced GPI. The first parameter was directly proportional to GPI whereas the product was inversely proportional to GPI. Moreover, a negative correlation (*R *= -0.42, p = 0.0069 for the 'no-correlation' null hypothesis) between the variables duration of algorithm application and average BG sampling frequency was found.

**Figure 7:**
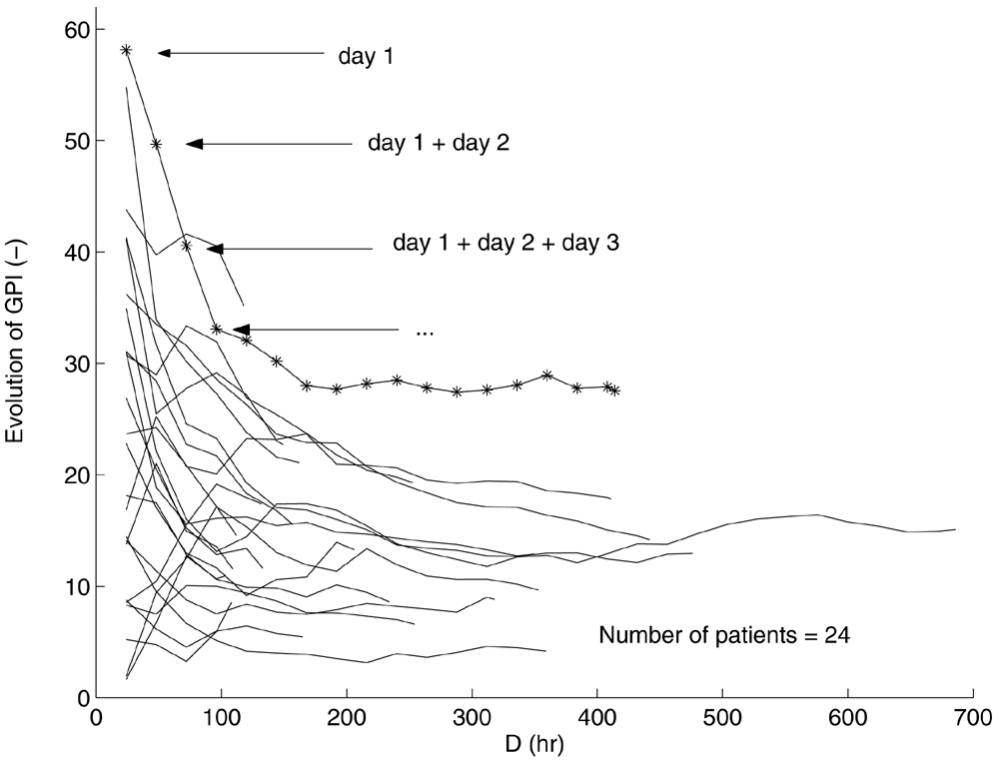
Evolution of the glycemic penalty index (GPI) as a function of duration of algorithm application. The evolution of the GPI values as a function of duration of algorithm application (*D*) for the patients of group 1 who stayed for at least 100 h in the intensive care unit (ICU). Each line represents a patient. For example, the first star represents the GPI value that is calculated based on the blood glucose (BG) observations of the first 24 h of that specific patient. The second star gives the GPI value based on the measured BG signal of the first 48 h; the third GPI value is computed based on the data of the first 72 h; etc. For the majority of the patients, a decreasing GPI trend was observed.

The impact of the duration of algorithm application on GPI is further clarified in Figure 7 for the patients (from group 1) who stayed for at least 100 h in the ICU. Every 24 h, the GPI was computed based on all previous BG observations of each particular patient. Each line of Figure 7 represents the GPI evolution of this specific patient as a function of the number of data (i.e., the time spent in the ICU) that were considered in the calculation process of GPI. For the majority of the patients, a decreasing GPI trend could be observed as more data (longer duration of the applied algorithm) were taken into consideration.

## Discussion

In this study we developed the GPI as a tool for assessing the dynamics of glycemic control in ICU patients. The designed formula returned a number between 0 and 100 with an 'ideal' level of 0 (indicating that all measured BG values fell within the normoglycemic target range) and a 'clinically acceptable' level of 23. Further, it was shown that GPI summarized the monitored glucose profile into one number more precisely than the traditional evaluation tools based on currently available clinical expertise. Finally, the average BG sampling frequency and the duration of algorithm application were found to be parameters that should be comparable for patient groups when comparing the performance of insulin titration algorithms.

### Mathematical computation of GPI

The developed GPI tool summarizes the level of tight glycemic control into a single number based on a grading system that scores low and high BG readings depending on their deviation from the target range. There are many advantages of GPI over the current standard methods. First, since both low and high BG readings are taken into account, GPI measures the overall BG dynamics. Since the assigned penalties are always absolute (positive), it is not possible that hypoglycemic and hyperglycemic penalties balance each other as can be the case when computing the average (morning) BG.

Second, only the BG readings that were effectively monitored are used in the GPI evaluation tool. Accordingly, unlike for area-under-the-curve methods, the GPI does not rely on any assumed (linear) relationship between measurements. While awaiting the creation of reliable near-continuous sensor devices for BG monitoring in an ICU setting, this is an important aspect as these assumed (linear) relations between observations do not necessarily approach the real (non-linear) blood glucose dynamics.

Third, a smooth penalty function (Figure 1) forms the basis of GPI leading to a gradual increase of the assigned penalties as the deviations from normoglycemia are enlarging. Measurement errors caused by sensor inaccuracies and methodology inaccuracies due to sampling handling have only a small level of influence on the assignment of the penalty, accordingly.

A fourth important feature of GPI is the independency of outlier measurements. Due to the imposed limits in the penalty function (if *BG *< 20 mg/dl or *BG *> 250 mg/dl, then β = δ = 100), extreme BG measurements (that may be related to sensor/methodology inaccuracies) cannot mislead the general algorithm assessment. Moreover, BG values lower or higher than these imposed limits would not lead to a clinically different treatment. This concept formed the basis of the specific region strategy in the error grid analysis for the evaluation of glucose sensors, as reported previously [32,33,43].

Finally, the computation of the relative contribution of the hypo- and hyperglycemic events to GPI allows us to further interpret the obtained GPI value. If we consider the BG profile of patient no. 1 (from patient group 1) as an example (see Table 4), based on the high GPI that was obtained (GPI = 49 > 23) it could be concluded that BG was poorly controlled in this patient. The relative contributions (expressed in terms of percentage) of the hypo- and hyperglycemic events (*C*_*Hypo *_and *C*_*Hyper*_, respectively) to GPI informed the clinician whether this non-optimal control behavior was caused by particularly low glucose events (if *C*_*Hypo *_> 75%), high glucose events (if *C*_*Hyper *_> 75%), or both (if *C*_*Hypo *_≈ *C*_*Hyper*_). The non-optimal performance of the algorithm for this patient example was mainly caused by the hyperglycemic events due to the large value for *C*_*hyper *_(see Table 4).

### Comparison of GPI with currently used methods

The computed GPI can be used to appropriately evaluate the level of tight glycemic control in a single patient based on clinical expertise. Existing methods may mislead an assessment, as is shown for patient no. 11 in Figure 4 (bottom panel). The average *morning *BG (103 mg/dl ≤ 120 mg/dl), the average BG (100 mg/dl ≤ 120 mg/dl), and the HGI (4 mg/dl ≤ 12 mg/dl) all suggest strict glycemic control whereas the GPI (27 > 23) denotes the less tightly controlled BG signal based on clinical expert knowledge. Both hypoglycemic and hyperglycemic events can be observed in the BG profile, which is further confirmed by the similarity between *C*_*Hypo *_and *C*_*Hyper *_for this patient.

Figures 2 and 3 summarize the assessment of the individual BG profiles by applying the existing methods and GPI. The shaded area is defined by the GPI 'clinical unacceptability' cut-off (*GPI *> 23) and the 'clinical acceptability' limits of the known techniques (average (morning) BG ≤ 120 mg/dl, HGI ≤ 12 mg/dl). In other words, the evaluation of the BG profiles of the patients belonging to this area may be misled by the existing methods (particularly the average morning BG and average BG, due to their high number of patients in the shaded areas and, to a lesser degree, the HGI). In fact, only few BG profiles were evaluated differently with HGI indicating that this method most approaches the clinical 'expert' GPI function. This also occurs in the high correlation coefficients for HGI and GPI.

The assessment of the performance of the BG algorithm on a population basis also depended on the selected technique. As observed in Table 4, the average morning BG (108 ± 20 mg/dl) and the average BG (114 ± 21 mg/dl) were below 120 mg/dl suggesting that the algorithm under study was adequate. The computed IQ ranges for the average (morning) BG, both below 120 mg/dl, confirmed this hypothesis. The computed IQ ranges of HGI and GPI, however, indicated that the applied algorithm did not result in clinically acceptable BG control for at least 25% of the patients. Indeed, a quarter of the HGI values were above 17 mg/dl (> 12 mg/dl) and a quarter of the GPI values were above 27 (> 23).

This study shows that the two most traditional measures (average morning BG and average BG) used for summarizing the overall glycemic control behavior, can mislead assessments of BG algorithms. This confirms the results described in [44]. Techniques that take into account the duration of hyperglycemia/hypoglycemia, such as HGI or the recently proposed 'notional duration of hyperglycemia/hypoglycemia' (i.e., the time since the observation of an abnormal BG till it returns to the accepted range [44]) may better indicate tight glycemic control. The GPI technique proposed in this manuscript, however, is explicitly founded on ICU expertise and may therefore be an alternative (or at least a supplemental) tool for adequately evaluating insulin titration algorithms in the ICU. The BG profiles of most individual patients were equally assessed using GPI and HGI, except for some patients as was illustrated in Figures 2 and 3 (shaded areas of the bottom panels).

A first weakness of the GPI is the non-consideration of the duration of hypoglycemic and hyperglycemic episodes since no (linear) relationship between discrete-time BG observations is assumed. Accordingly, the algorithm assessment may be misleading, as the number of intermittent BG measurements (and the number of assigned penalties) can typically be higher with unstable BG behavior (i.e., BG observations outside the normoglycemic target range). Only area-under-the-curve measures (like HGI) can potentially take into consideration the duration of these glycemic deviations under the assumption that the imposed (linear) relationship between the measurements approaches the real blood glucose dynamics. Moreover, the duration of deviating episodes can only be precisely taken into account with a reliable and accurate near-continuous glucose sensor. The use of such a device for the evaluation of a BG algorithm even allows us to label the GPI measure as area-under-the-curve method (that incorporates the duration of glycemic deviations), since then a penalty is 'continuously' assigned to each BG (measured at each time instant, e.g., every minute) and since hypoglycemic and hyperglycemic deviations cannot balance each other. While awaiting reliable near-continuous glucose sensors [15,19,45], it is advised to sample BG at fixed time intervals (e.g., every hour or every 2 h for the duration of the study) to minimize the effect of the current weakness.

A second weakness of GPI is the ignorance of the severity of extreme (but exceptional) BG measurements due to hypo- and hyperglycemic cut-off values (e.g., δ_300 mg/dl _= δ_450 mg/dl _= 100). Though the reasons for using these cut-off values are well founded (see above), we advise counting the number of alarm BG observations (i.e., BG < 40 mg/dl [35] and BG > 200 mg/dl [8]) to better interpret the obtained GPI.

Previous studies have already indicated the relationship between improved clinical outcomes on the one hand and reduced average morning BG [8,9] and reduced HGI [28] on the other. It is important to note that the relationship between GPI and clinical outcome has not been shown yet. The design of GPI is purely founded on currently available clinical expertise. Future studies are necessary to verify whether low GPIs effectively correspond to reduced mortality and morbidity, which is however expected from a clinical 'expert' point of view and from the high correlation between GPI and HGI.

### Weight determination for the selected parameters

The BG sampling frequency in the insulin titration guidelines used in patient group 1, varied as a function of the level of glycemic control. When the blood glucose was more difficult to control (unstable glucose dynamics), more frequent sampling occurred. The full patient data of group 1 comprised the initial (unstable) and more chronic (stable) phase of each patient's stay in the ICU. An increasing duration of algorithm application (which implicitly indicates a longer stay in the ICU, typically associated with more stable glucose dynamics) artificially improved the average overall BG control behavior leading to lower GPIs (see second panel of Figure 6). Figure 7 additionally clarifies the relationship between GPI and duration of algorithm application. The GPI decreases when more data (i.e., longer time/duration in the ICU) are considered in its computation process.

Further, this increasing duration of algorithm application lowered the average BG sampling frequency (expressed in the negative correlation between duration of algorithm application and average BG sampling frequency) since less BG observations were required in the chronic 'stable' period (due to the nature of the used protocol). Therefore, the first panel of Figure 6 that illustrates the relationship between tight glycemic control (low GPI) and a low average BG sampling frequency is explained by the long time that the algorithm was applied to the patients of group 1. In case the duration of algorithm application was kept constant and limited (only the first 48 h after admission), an increase of the BG sampling frequency resulted in more strict BG control (lower GPI) as depicted in the top panel of Figure 5. It can be concluded that both duration of algorithm application and average BG sampling frequency are two important parameters that should be taken into consideration when assessing or comparing different BG control algorithms.

### Practical use

For the design of future studies that compare the performance of different insulin titration algorithms applied to critically ill patients, we encourage other research groups to rely on the 'similarity' condition: the duration of algorithm application and the BG sampling frequency should be similar in patient groups. We further encourage other groups to consider GPI as supplemental tool to other advanced measures (e.g., HGI, 'notional duration of hyperglycemia/hypoglycemia') besides more traditional measures (e.g., average morning BG, average BG) for adequately assessing the overall level of (BG) control.

## Conclusion

The use of nurse-driven BG control algorithms is becoming standard practice in ICUs. New (semi-automated) insulin titration algorithms are currently under development but require an appropriate evaluation before accepting them as state-of-the-art. In this study, we presented the computation of the GPI as a tool to compare different BG control algorithms. This index encompasses the overall BG dynamic behavior per patient in a single number based on clinical expertise. The method is affected by BG sampling frequency and duration of algorithm application, which should be similar for adequate comparison of these algorithms.

## Key messages

The Glycemic Penalty Index (GPI) encompasses the overall BG dynamic behavior per patient in a single number. It is the average of all penalties that are individually assigned to each measured BG value based on an optimized smooth penalty function.

The computation of GPI returns a number between 0 (no penalty) and 100 (the highest penalty).

The computation of GPI may be an alternative or supplemental tool to evaluate and to compare the performance of BG algorithms.

A higher BG sampling frequency and a longer algorithm application duration resulted in an apparently better performance of the insulin titration algorithm, as indicated by a lower GPI.

The BG sampling frequency and the duration of algorithm application should be similar when comparing algorithms.

## Abbreviations

BG = blood glucose; GPI = glycemic penalty index; HGI = hyperglycemic index; ICU: intensive care unit.

## Competing interests

The authors declare that they have no competing interests.

## Authors' contributions

TVH designed the engineering study, developed the GPI concept, and wrote the manuscript. JDB and MB helped to statistically analyze the data and to draft the manuscript. BDM conceived of the engineering study, and participated in its design and coordination and helped to draft the manuscript. GVdB designed the clinical study, participated in the design of the engineering study and helped to draft the manuscript. All authors read and approved the final manuscript.
